# Parental Health Literacy as a Determinant of Parenting Practices and Early Childhood Health Outcomes: A Systematic Review

**DOI:** 10.3390/children13050685

**Published:** 2026-05-16

**Authors:** Melinda Csima, Henrietta Bánfai-Csonka, Viktória Keresztes, Judit Podráczky, Evelin Soós, Judit Fináncz

**Affiliations:** 1 Institute of Education, Hungarian University of Agriculture and Life Sciences, 7400 Kaposvár, Hungary; keresztes.viktoria@uni-mate.hu (V.K.); podraczky.judit@uni-mate.hu (J.P.); soos.evelin@uni-mate.hu (E.S.); 2 MTA-MATE Early Childhood Research Group, 7400 Kaposvár, Hungary; 3 Faculty of Health Sciences, University of Pécs, 7621 Pécs, Hungary; csonka.henrietta@pte.hu; 4 Education and Society Doctoral School of Education, University of Pécs, 7624 Pécs, Hungary

**Keywords:** health literacy, parents, early childhood, health outcomes, parental practices, systematic review

## Abstract

**Highlights:**

**What are the main findings?**

- Parents’ health literacy links to effective childcare and parenting practices.
- Higher parental health literacy increases parental responsibility.

**What are the implications of the main findings?**

- Gaps in measuring parental health literacy underscore the need for valid tools.
- Enhancing parental health literacy through education may reduce health inequalities.

**Abstract:**

**Objectives**: Parental health literacy plays a pivotal role in shaping caregiving practices and influencing their children’s health. The aim of this systematic review is to analyze studies examining health literacy among parents raising children under five, in relation to caregiving practices and children’s health outcomes. **Methods**: The research was conducted in accordance with PRISMA protocol. The sample of the study was determined by using the terms related to “health literacy” AND “parent” (OR “caregiver” OR “mother” OR “father”) AND “child” AND “measure” (OR “instrument” OR “tool” OR “questionnaire” OR “survey” OR “interview”) in ERIC, PUBMED, Scopus and WOS databases published between 2015 and 2024. **Results**: Of the 1726 results identified, 18 studies met the inclusion criteria. The reviewed studies on parental health literacy place particular emphasis on oral health literacy, nutrition/food literacy, vaccine literacy, and fever literacy. **Conclusions**: Based on the included studies, parents’ health literacy is generally found to be significantly associated with the adequate fulfilment of children’s care needs, the implementation of appropriate parenting practices, and the effective management of childhood illnesses (although findings regarding vaccination willingness are inconsistent). Overall, these associations may carry long-term implications for children’s health.

## 1. Introduction

Major developments in the fields of medicine and health sciences have contributed to notable improvements in both life expectancy and healthy lifespan [1]. Leading causes of death are increasingly chronic degenerative diseases that can be influenced by lifestyle. The LaLonde report (1974) has already highlighted the importance of lifestyle, resulting in an increasing emphasis on prevention and the responsibility of the individual to shape their own health, with a corresponding increase in health-related knowledge and competences [2]. In this context, the term health literacy (HL) emerged in 1974 [3]. Since then, several attempts have been made to define it precisely, among which Nutbeam’s (p. 263) definition has become the most widely used: “health literacy refers to the personal, cognitive and social skills which determine the ability of individuals to gain access to, to understand, and use information to promote and maintain good health” [4].

In parallel with the consensus interpretation of the concept, the development of health literacy measurement tools began, and as a result, population health literacy surveys were launched [5]. Initially, the basic skills required for the interpretation of health-related topics were assessed through various tests. At first, the REALM test (Rapid Estimate of Adult Literacy in Medicine) became widespread, which focused on measuring functional health literacy [6]. Another frequently used instrument was the TOFHLA (Test of Functional Health Literacy in Adults) and its abbreviated version, S-TOFHLA, which tested the understanding of terms encountered in health care [7]. The Newest Vital Sign (NVS) questionnaire, developed in 2005, measures functional health literacy based on reading comprehension and numeracy skills [8]. Based on previous measurement experiences, the most widely used measurement tool today, the HLS-EU-Q, was developed within the framework of The European Health Literacy Survey, which measures health literacy-related competencies in the areas of prevention, health care system and health promotion, and captures all three dimensions of health literacy, including functional, communicative, and critical health literacy [9].

Nowadays, in addition to health science, other disciplines also consider it the subject of their research, thus giving it a raison d’être in educational research as well. In the educational discourse, the study of early childhood influences has received increasing attention over the past two decades [10], of which the formation of health-related habits is of particular importance [5].

These studies have shown that the health-related knowledge, health behavior, and caregiving practices of the adults surrounding the child in early childhood are decisive for the child’s later health outcomes. During this period, the influence of parents is more dominant than institutional education in terms of decisions affecting health and the formation of health-promoting habits, as children spend more time in the family environment than at later ages. In connection with early parenting practices, previous studies primarily explored parents’ practices related to safety practices (especially sleeping position, car seat use, fire safety, smoking), feeding practices, development promotion, and health care utilization practices [11,12]. In addition to caregiving practices, the model conveyed by parents and their health-related decisions are also determining factors in the conscious formation of the child’s health-promoting habits. Research findings on early parenting practices have led to an increasing focus on parents with young children as a specific target group, in addition to health literacy studies of the general population [5,13,14].

Despite the fact that understanding the health literacy of parents raising young children is of paramount importance for children’s health status and quality of life, its accurate measurement presents substantial methodological challenges for researchers. Numerous studies apply general population health literacy instruments (such as HLS-EU-Q, TOFHLA, or NVS) in parental populations as well. However, these tools do not include content specific to parenting responsibilities, including knowledge related to childcare practices and childhood diseases. This highlights the need to distinguish between parents’ general health literacy (“parents’ health literacy”) and the more specific form of health literacy directly associated with the parental role (“parental health literacy”).

Within the early childhood period, particular attention is paid to young children under 5 years. The reason for this is, on the one hand, that in many countries, compulsory entry into the institutional education system is linked to this age [15], and on the other hand, international professional organizations present children under five as a separate group when publishing morbidity and mortality statistics [16,17]. Therefore, understanding the health literacy of parents raising children under 5 years of age can be considered of paramount importance. Accordingly, *the aim of our systematic review is to analyze studies examining the health literacy of parents raising children under the age of five, in relation to caregiving practices and children’s health outcomes.*

Based on the PICO (2024) format, the following research questions were used in our study [18]:

1. In what interpretive frameworks do the examined studies place parents’ health literacy?
2. What measurement instruments are used to assess parental health literacy?
3. When examining parental health literacy, which aspects do the studies focus on regarding care practices and child health?
4. What correlations do the studies identify between parental health literacy, childcare practices, and child health?

Answering the above questions can contribute to exploring the contexts of parental health literacy research and identifying challenging issues. They can help to identify areas of parenting competence that need to be developed in relation to young children’s health development, care and health outcomes.

## 2. Materials and Methods

Our study was conducted following the principles of the Preferred Reporting Items for Systematic Reviews and Meta-Analyses (PRISMA) 2020 protocol [19]. The study protocol has PROSPERO registration, whose identifier is: CRD42024592792.

### 2.1. Information Sources and Eligibility Criteria

Four databases, including ERIC, PUBMED, Scopus and WOS were systematically searched using terms related to “health literacy” AND “parent” (OR “caregiver” OR “mother” OR “father) AND “child” AND “measure” (OR “instrument” OR “tool” “questionnaire” OR “survey” OR “interview”). All search terms are outlined in Table 1.

The inclusion criteria for the systematic review were defined as follows: only peer-reviewed empirical studies published in English-language scientific journals between 2015 and 2024 were considered. Eligible studies focused on the health literacy of parents raising children under the age of five and examined issues related either to children’s health outcomes or to parental caregiving practices. Studies were excluded if they were review articles, study protocols, pilot studies, policy papers, or validation studies of measurement tools without reporting empirical results. Additional exclusion criteria included studies focusing on children with special educational needs or chronic diseases, studies without findings related to parental health literacy, and studies in which parental health literacy was not examined in connection with childcare practices or children’s health needs and outcomes. Studies whose full texts were unavailable were also excluded from the review (Table 2).

### 2.2. Selection Process

In the first phase of the search, ERIC database recorded 53 results, PUBMED found 249 results, SCOPUS database identified 1095 studies, while WOS detected 326 papers. In addition, three further sources were identified through supplementary search procedures. All data was imported into a Microsoft Excel spreadsheet, where out of a total of 1726 results, 492 duplicates were removed. From the remaining database of 1234 bibliographic data, we excluded 594 based on title review, leaving 640 sources. After that, 390 studies were excluded after abstract review, leaving 250 full-text studies, of which 18 studies met the criteria.

During the study selection process, a multi-stage screening procedure was applied. In the first stage, titles and abstracts were independently screened by four researchers (MC, JF, VK, and ES). Each record was assessed by two independent reviewers in parallel to ensure consistency and reduce selection bias. Prior to screening, reviewers agreed on the application of the predefined inclusion and exclusion criteria to standardize decision-making. In cases where discrepancies arose during the title and abstract screening phase, disagreements were first discussed among the reviewing pairs. If consensus could not be reached, a fifth senior researcher (JP) was consulted to make the final decision regarding study eligibility.

In the second stage, full-text articles were retrieved and independently assessed for eligibility by two reviewers (MC and JF). Full-text screening followed the same predefined eligibility criteria used in the initial screening phase. Any disagreements at this stage were resolved through discussion between the two reviewers; if consensus was not achieved, a third reviewer (JP) made the final decision.

**Figure 1:**
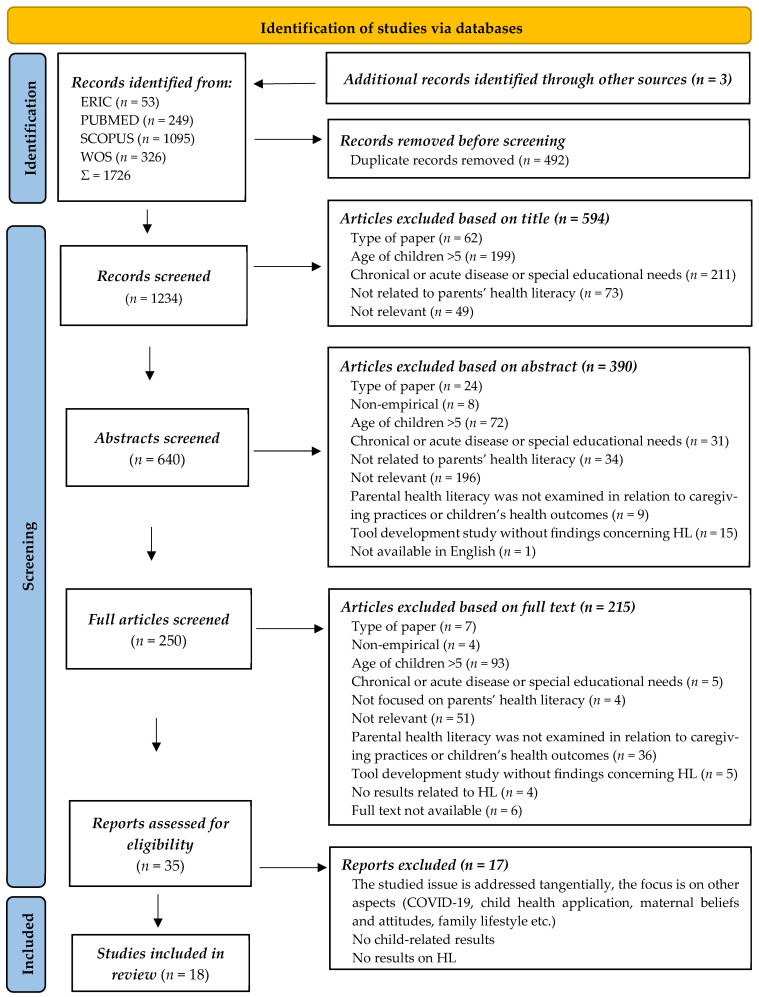
Flow chart of the searching process according to the PRISMA protocol.

This multi-reviewer process ensured methodological rigor, reduced selection bias, and enhanced the reliability of the study inclusion procedure. The individual steps of the selection process are illustrated in the PRISMA flow chart (Figure 1).

### 2.3. Data Synthesis

Due to the heterogeneity of study designs, outcome measures, and assessment tools, a meta-analysis was not feasible. Therefore, the findings were synthesized narratively following the Synthesis Without Meta-analysis (SWiM) reporting guideline [20]. Studies were grouped according to the measurement tools used and their main content areas. The results were then summarized by considering the direction and characteristics of the observed associations across studies. Where applicable, greater emphasis was placed on findings supported by multiple studies or consistent patterns across different contexts.

### 2.4. Quality Assessment

For critical appraisal and to reduce the risk of bias, each included study underwent a structured quality assessment. The Newcastle–Ottawa Quality Assessment Scale adapted for cross-sectional studies was applied to evaluate the risk of bias in cross-sectional studies [21]. Studies were assessed across three domains—selection, comparability, and outcome—and classified as satisfactory, good, or very good. The Newcastle–Ottawa Quality Assessment Scale for cohort studies [22] was used to assess risk of bias in cohort studies. Finally, quasi-experimental studies were appraised using the JBI critical appraisal tool [23]. The results of the quality assessment are presented in the Supplementary Materials (Tables S1–S3) and were considered in the interpretation of the findings during the narrative synthesis.

## 3. Results

### 3.1. Characteristics of the Included Studies

The main characteristics of the 18 selected studies are summarized in Table 3. Although the concept of health literacy has been interpreted multidisciplinary since the 1990s [24], the studies identified during the selection process were primarily published in journals within medical and health sciences fields. Of the included studies, only one [25] appeared in a journal that, according to SCImago data, can be classified under education category, too.

Seven of the studies were published during the last five years (2020–2024), 11 studies before 2020 (2015–2019). More than a third of the studies (*n* = 7) are based on research conducted in the USA, in addition, two studies contain results from Germany and one each from different countries (Australia, Brazil, The Netherlands, Israel, Turkey, Iran, India, Myanmar, Sweden).

The included studies examine parental health literacy mostly in relation to feeding (*n* = 4) and vaccination (*n* = 3), but in addition to general child health (*n* = 1), other areas also appear, such as children’s dental health (*n* = 2), fever management (*n* = 2), mental health (*n* = 1), weight control (*n* = 1), early parenting practices (*n* = 1), second-hand smoke exposure (*n* = 1), allergy prevention (*n* = 1), and diarrhea prevalence (*n* = 1) (Table 3).

### 3.2. Measuring Tools of Parental HL

The majority of the studies included in the analysis (*n* = 15) measured parents’ health literacy by assessing general health literacy, mostly using standardized self-administered questionnaires. The most widely used measurement tool was Newest Vital Sign (*n* = 5), but several studies also used different versions of the REALM (*n* = 2) and HLS-EU (*n* = 3) questionnaires (Table 4). In addition, a few studies used self-developed (*n* = 2) or adapted (*n* = 3) questionnaires. Some studies examined health literacy related to a specific area, such as oral HL (*n* = 2) and hygiene HL (*n* = 1) (Table 3).

Considerable heterogeneity was identified in the measurement tools applied across the included studies. Several different instruments were used to assess parents’ health literacy, making direct comparison of findings more difficult. At the same time, tools specifically developed to measure parental health literacy did not appear to be widely established in the literature.

### 3.3. Core Content Areas of the Included Studies

#### 3.3.1. Feeding

In line with the specificities of the age group, nutrition, especially breastfeeding, receives special attention in two studies. In their study, Hosseini et al. [31] investigated the relationship between maternal health literacy and breastfeeding duration among Iranian primiparous women. Their results confirmed a significant association between the variables studied: higher levels of health literacy were associated with longer duration of breastfeeding. This is contradicted by the results of Graus et al. [29], who, in their longitudinal cohort study conducted in Germany, found no association between maternal health literacy and exclusive breastfeeding of the child until the age of four months.

Heerman et al. [30] examined the association between parental health literacy and more permissive feeding practices in a Latino population. Their results showed that lower levels of parental health literacy were associated with more permissive, unhealthy parent feeding practices, which are associated with a higher risk of childhood obesity.

Northrup and Smaldone [38] examined the attitudes, norms and meal selections behaviors of mothers with 2–3-year-old children regarding nutrition. Findings showed that approximately one third of mothers had limited health literacy, which was manifested in several areas: they chose larger than recommended food portions for their children and were less likely to correctly identify body silhouettes of overweight children on pictograms.

#### 3.3.2. Vaccination

Many countries provide compulsory and optional vaccines to prevent specific communicable diseases, which are typically administered in early childhood. Legislation on compulsory vaccination varies considerably from country to country, resulting in different levels of vaccination coverage for certain diseases [16,17]. Whether children receive recommended but not mandatory vaccinations is significantly influenced by parents’ views and beliefs about vaccination. This was examined in the context of parental health literacy by Amit Aharon et al. [27], who explored the background of decision-making regarding vaccinations in their Israeli study. Examining functional, communicative, and critical health literacy, the authors found that higher levels of parental health literacy and greater trust in informal information sources were directly correlated with lower willingness to vaccinate. In contrast, in the study by Johri et al. [32] among Indian mothers, a statistically significant crude association was observed between health literacy and DTP3 (three-dose diphtheria, tetanus, and pertussis) completion among mothers with medium and high levels of health literacy: children of mothers with higher levels of health literacy received all three DTP vaccines at a higher rate.

The study by Meppelink et al. [37] investigated in the Netherlands whether biased selection and evaluation of online health information occur in the context of early childhood vaccination, and to what extent parents’ levels of health literacy inhibit or facilitate these cognitive biases. Based on their findings, parents of young children were more likely to select information that was consistent with their pre-existing beliefs, which they perceived as more credible, useful, and persuasive. Both biased information selection and biased perception of message credibility were more prevalent among parents with higher levels of health literacy.

#### 3.3.3. Oral Health

Tooth decay is one of the major health issues in early childhood causing pain and adversely impacts the quality of life in young children. It is noteworthy that even in advanced economies, the rate of untreated dental caries is significant for the studied age group [44]. The level of parental health literacy greatly influences decisions about taking one’s children to dental care, which is also confirmed in relevant studies [45].

Brega et al. [25] explored the relationship between children’s oral health outcomes and parental health literacy among Navajo Nation families. According to their findings, parents with higher health literacy levels were more likely to view their children’s oral health as their personal responsibility. Moreover, stronger health literacy was linked to an increased perception of oral health issues and a greater appreciation for the benefits of recommended oral hygiene practices.

Menoncin et al. [36] explored the relationship between parental oral health literacy and the utilization of pediatric dental services. Their findings indicated that higher parental oral health literacy was significantly associated with increased utilization of dental care among children.

#### 3.3.4. Fever

Fever is an accompanying symptom in a significant proportion of illnesses that occur in early life, so it is important to understand parents’ knowledge and practice regarding fever treatment, as inadequate fever management can affect the course of the illness and the child’s well-being. Due to the results of medical and health science research, professional protocols related to fever management have changed in recent years [46], about which in many cases lay parents do not have adequate information or they adhere to previously considered proven practices. Recognizing this, numerous studies are aimed at exploring parents’ beliefs, knowledge and practices related to fever [47,48].

Alqudah et al. [26] compared fever management practices and knowledge of parents with different levels of health literacy in Australia. Their results showed that regardless of health literacy level, parents had limited knowledge and poor practices in relation to fever. In contrast, the results of the study by Menekşe et al. [35] showed a positive, linear relationship between the level of parental health literacy and fever management: in the case of more favorable parental health literacy, the scores on the Parents’ Fever Management Scale proved to be higher.

#### 3.3.5. Additional Areas in the Studies

In addition to the aforementioned areas, the studies also focused on several other domains that hold critical importance for early childhood health. Mekhail et al. [34] examined the effects of an intervention delivered through extended home visits among socioeconomically disadvantaged parents in Sweden. Their results showed no correlation between the level of parental health literacy and the health outcomes of the children examined. In the study by Lee et al. [11], the correlations between maternal health literacy (MHL), parenting self-efficacy and early parenting practices (Early Parenting Practices Index—EPPI) were examined among low-income families. Maternal health literacy (MHL) demonstrated a positive correlation with parenting self-efficacy and early parenting practices, including domains such as safety, feeding, development promotion, and health care utilization. Although the association between early parenting practices and health literacy did not reach statistical significance, a trend was observed suggesting that mothers with adequate health literacy engaged in more informed and guideline-consistent parenting behaviors.

Liechty et al. [33] focused on a different area in relation to parental health literacy within the framework of the STRONG Kids longitudinal panel study: they examined parents’ use of weight control strategies. The findings indicate that health literacy influences parental perceptions of child weight management strategies and their preferences for seeking health-related information.

In their study, Welkom et al. [41] examined the associations between health literacy and child second-hand smoke exposure among caregivers of Head Start children. Their results indicate that caregivers with lower health literacy exhibited higher levels of home air nicotine and child salivary cotinine, indicating greater exposure to second-hand smoke. Additionally, lower parental health literacy was associated with stronger endorsement of negative smoking expectancies.

In recent decades, the number of people with diagnosed allergies has increased dramatically, the symptoms of which often appear in childhood and can often be traced back to environmental influences during childhood. Examining the associations between parental health literacy and allergy prevention behaviors is particularly relevant, as a paradigm shift has occurred in the field of allergy prevention in recent years [49], about which parents are often insufficiently informed. The study by Pawellek et al. [39] draws attention to the fact that mothers with lower health literacy are more likely to have allergen avoidance behavior (feeding hypoallergenic infant milk, avoiding specific foods in the first year, measures to reduce dust mite exposure) and are less likely to have exclusively breastfed their child in the first four months.

Diarrheal infectious diseases are particularly prevalent in early childhood, which can be particularly dangerous for the immature immune system and, if not treated appropriately, can have fatal consequences. In this regard, Soe et al. [40] examined the association between hygiene practices and childhood diarrhea among Myanmar families with children under five. Their results showed that the low level of household hygiene service (regarding to father’s hand washing practices, water collection and toilet facilities, house floor type) and hygiene health literacy of parents significantly increase the risk of childhood diarrhea.

In addition to factors related to somatic health, the mental dimensions of health are less emphasized in this age group: only one study focusing on mental health issues could be included in the analysis. Cormier et al. [28] examined the relationship between parental eMental health literacy and their children’s mental difficulties. Parents with lower eMental health literacy were more likely to perceive their own child as belonging to a higher risk group for mental health disorder and less likely to seek professional help for perceived mental health problems.

### 3.4. Results of Quality Assessment

The included studies were systematically appraised for methodological quality, and the results of this assessment are presented in the Supplementary Materials (Tables S1–S3). This evaluation provides an overview of the overall risk of bias across the evidence base. For both cross-sectional and cohort studies, the Newcastle–Ottawa Quality Assessment Scale was used to evaluate the risk of bias [21,22]. In cross-sectional studies, assessments were conducted across three domains—selection, comparability, and outcome. Based on this evaluation, seven of the 14 studies were classified as satisfactory, four as good, and three as very good (Table S1). The two cohort studies were both rated as being of fair methodological quality (Table S2). In addition, the two quasi-experimental studies were appraised using the JBI critical appraisal tool [23], and both were assessed as having a low risk of bias (Table S3).

The results of the quality assessment were considered in the interpretation of the findings. No studies were excluded based solely on quality ratings, as the aim of the review was to provide a comprehensive overview of the available evidence. Overall, the majority of included studies were of moderate to good quality, which supports the robustness of the synthesized findings, although some caution is warranted due to variability in study design and measurement approaches.

### 3.5. Synthesis of the Main Findings Based on the Included Studies

**Figure 2:**
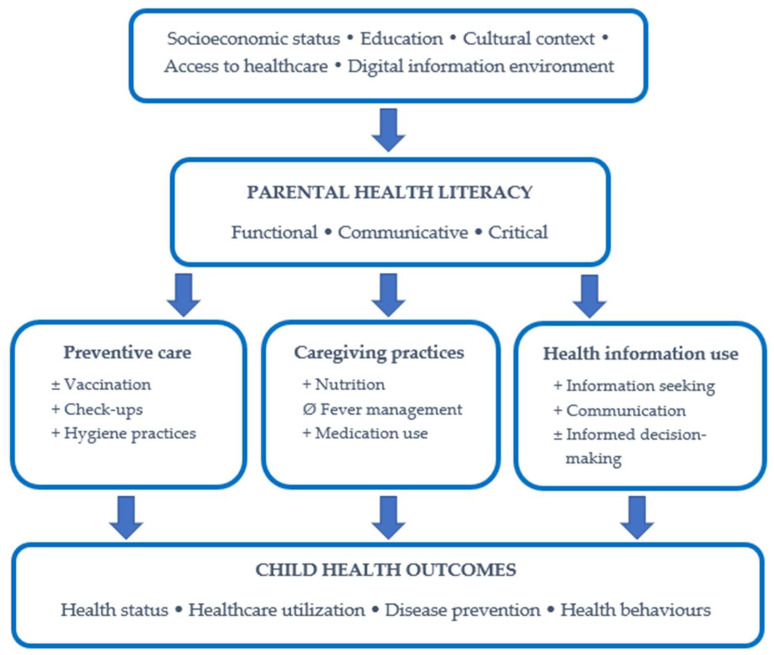
Synthesis of the Associations Between Parental Health Literacy and Child Outcomes. Note: + positive association; ± inconsistent association; Ø no association.

Across the included studies, parental health literacy was associated with several dimensions of child health and caregiving practices, although the direction and strength of these associations varied across outcome domains. The conceptual figure presents (Figure 2) an integrated overview of the main associations between parental health literacy and child health outcomes.

Parental health literacy influences children’s health status through several mediating mechanisms, primarily including preventive health behaviors, caregiving practices, and the processing of health information and decision-making. Based on the findings, higher levels of health literacy were generally associated with more favourable caregiving and preventive behaviors. However, inconsistent associations were observed in certain areas, particularly in relation to vaccination-related decisions.

## 4. Discussion

The primary goal of our systematic review was to examine the health literacy of parents raising young children in relation to parenting practices and their children’s health outcomes. Parents constitute a key group of the adult population in this regard, as they are responsible not only for their own health, but also for that of their children.

There are numerous studies examining parents’ health literacy across various regions and sociodemographic groups. However, the available evidence remains difficult to compare because of the considerable heterogeneity in measurement approaches. Most existing studies rely on instruments originally developed for the general population, which may limit the extent to which findings capture the specific competencies required in child-rearing and caregiving contexts. Although some attempts have been made to develop more parenting-specific tools [50], their adoption in empirical research has remained limited. These methodological inconsistencies highlight the need for a more precise operationalization of parental health literacy and for measurement approaches that better reflect the realities of parenting practices. Strengthening the conceptual framework of parental health literacy could improve the comparability of future studies and contribute to a clearer understanding of how parental competencies influence children’s health and wellbeing. Given its potential impact on parenting behaviors, healthcare utilization, and the home environment, parental health literacy should be considered an important determinant of long-term child health outcomes [14].

Examining parents’/parental health literacy, mothers are primarily at the forefront, presumably due to traditional parental roles on the one hand, and on the other hand, in many cultures, fathers are less involved in childcare tasks in the early years of life. The limited number of studies investigating fathers’ health literacy show lower levels compared to mothers [51]. Based on the WHO definition of health literacy [52], Jiregna et al. (p. 145) defines maternal health literacy as follows: “pertains to a mother’s capacity to obtain, understand, appraise, and apply information regarding maternal and child healthcare throughout her pregnancy, childbirth, and postpartum period” [13].

In measuring parents’ health literacy, numerous studies have focused on parents with a low socio-cultural background [53,54], using the term literacy in a broader sense, with an emphasis primarily on basic skills such as reading comprehension and numeracy. The measurement tools used in these studies typically targeted parents with low levels of education (less than 7th–8th grade), using informational materials related to health and illness. In connection with these, the adequacy and effectiveness of leaflets, patient education materials, and drug labels were examined. In relation to low levels of education, multiple studies highlight the occurrence of medication dosing errors and their possible implications [55,56].

Numerous studies have been conducted in pediatric care and emergency care settings, examining the justification of service utilization (e.g., in cases of fever or accidents) [26,57].

We found several studies that examined parents’ health literacy in relation to a specific disease or developmental disorder [58,59], and some published results exploring a specialized topic with limited scope (e.g., honey consumption) [60].

In addition, many studies have addressed inequalities in access to health information [61,62] and, in this context, the issue of digitalization. Furthermore, children’s screen time and physical activity were examined in relation to parental health literacy [63]. Most of the studies mentioned above did not meet our selection criteria (age group, lack of correlation analyses, etc.). Several studies were excluded during the selection process because they examined parental health literacy isolated without looking for a relationship between early parenting practices or child health outcomes [64,65].

In assessing parents’ health literacy, some studies focused on a specific area—such as oral HL [25], nutrition or food HL [66], fever literacy [67], foot HL [68], and hygiene promotion HL [40]; however, the aim was not always to explore the correlations. Several studies focused on the validation of a measuring instrument—developed by the authors—without presenting the results regarding parents’ health literacy [51,69].

According to the inclusion and exclusion criteria, 18 studies were included in the analysis. In the examined field, cross-sectional studies predominate; however, measurements have also been conducted as part of longitudinal studies, with partial results published in separate papers, which meet the inclusion criteria of our systematic review (Table 4).

The papers included in the analysis focused on different areas depending on the age of the children (Table 3): in the case of infants, parents’ health literacy was studied primarily in relation to breastfeeding, complementary feeding, and vaccination. For toddlers and preschoolers, a broader spectrum of topics appears: in addition to hygiene-related practices (e.g., hand washing), parental health literacy was examined in relation to other parental practices (e.g., fever management, secondhand smoke, nutrition) and health outcomes (e.g., oral health, use of child health care).

Based on the findings of the included studies, it cannot be clearly stated that higher parental health literacy is always more beneficial for the child, although the above trend mostly prevails.

In the context of feeding practices, the majority of studies consistently report that parents with higher health literacy place greater emphasis on their children’s nutrition [30,38]. At the same time, the findings indicate a more nuanced pattern, as some studies highlight the role of additional factors such as parental stress and health literacy in shaping feeding behaviors, including an increased risk of unhealthy feeding practices and childhood obesity. Moreover, lower maternal health literacy was associated with a higher tendency to adopt allergen-avoidant behaviors and a reduced likelihood of exclusive breastfeeding, although the evidence regarding breastfeeding outcomes was not fully consistent across studies.

Moreover, higher parental health literacy has been associated with an increased sense of personal responsibility for children’s oral health [25]. In this context, parents with limited health literacy demonstrated significantly poorer oral health status and reported lower oral health-related quality of life for their children. Furthermore, lower levels of parental oral HL were associated with a reduced likelihood of children attending preventive dental visits. A similar correlation can be observed in the area of second-hand smoking [41]: children of parents with lower health literacy are more exposed to the negative effects of smoking.

In contrast, contradictory results have been obtained in the area of vaccination: some studies highlight an association between higher levels of health literacy and biased information selection [27,37], which may contribute to reduced willingness to vaccinate. However, Johri et al. [32] demonstrated in their study that children of mothers with higher health literacy were more likely to receive age-appropriate vaccinations. The inconsistent findings regarding vaccination-related behaviors may reflect the multidimensional nature of health literacy. While higher functional health literacy may facilitate access to and understanding of vaccination-related information, higher critical health literacy may also encourage parents to question medical recommendations and seek alternative sources of information. Cultural and healthcare system differences may further contribute to the variability of findings across studies, as trust in healthcare professionals and public health institutions differs substantially between settings. These findings suggest that the relationship between parental health literacy and vaccination behaviors is complex and does not consistently operate in a protective direction.

In addition, the studies identified an area in which parents demonstrated a substantial lack of information, regardless of their health literacy level. This pertains to fever literacy, where frequent changes in fever management protocols have resulted in parents not having adequate knowledge [26].

Beyond caregiving practices, previous health literacy studies and reviews have also demonstrated associations between limited health literacy and adverse health-related outcomes, including increased healthcare utilization, hospital readmissions, and emergency department use [70,71]. These findings further support the relevance of parental health literacy, as caregiving decisions and health management practices during early childhood may also contribute to longer-term child health outcomes and patterns of healthcare use, particularly in situations involving more complex care needs.

## 5. Study Limitations

As a limitation of our review, we included only peer-reviewed journal articles published in English and retrieved exclusively from WOS, Scopus, PubMed, and ERIC databases. Consequently, relevant studies may have been omitted due to the language restriction and the limitations inherent in our keyword strategy. Moreover, cross-country differences in healthcare and social service systems, as well as variations in cultural contexts, may influence parental health literacy and the thematic focus of health education practices examined in the included studies. Finally, most studies assessed parental health literacy using instruments designed to measure general HL, while tools specifically developed for evaluating parental health literacy were applied only sporadically, potentially limiting the comparability and depth of available evidence.

## 6. Conclusions

During the study period (2015–2024), numerous studies examined parents’ health literacy, primarily focusing on general health literacy among parents. In contrast, considerably fewer studies addressed parental health literacy as a distinct construct related to parenting roles and responsibilities, such as caring for and managing the health needs of young children.

Based on the reviewed studies, parents’ health literacy is associated with several aspects of childcare and health-related parenting practices, including the effective management of childhood illnesses, preventive behaviors, nutrition-related practices, and oral health behaviors. Nevertheless, the findings also indicate that these relationships are complex and may vary across specific health domains and behavioral contexts.

The results further demonstrate the increasing emergence of domain-specific forms of health literacy, particularly oral health literacy, nutrition and food literacy, vaccine literacy, and fever literacy among families with children aged 0–5 years.

Most studies identified in this review originated from the fields of medicine and health sciences, while the educational dimensions of parental health literacy received comparatively limited attention. Given the important role of early childhood educators in supporting health-promoting behaviors and parental guidance, stronger integration between health literacy research and educational sciences appears warranted.

Overall, this systematic review contributes to a broader understanding of the relationship between parental health literacy, caregiving practices, and child health outcomes, and may support the development of future interventions aimed at promoting parental health literacy and child well-being.

## 7. Future Directions

Understanding the characteristics of parental health literacy and caregiving practices is essential for advancing child health outcomes. The findings of our systematic review highlight conceptual ambiguities and methodological shortcomings in the measurement of parental health literacy, reflecting heterogeneity in definitions, operationalization, and assessment approaches across studies. These limitations underscore the need for the development of valid, reliable, and theory-based measurement tools that can adequately capture the special characteristics of parental health literacy. Future research should therefore focus on developing standardized assessment instruments specifically targeting caregiving-related competencies, such as managing the health needs of young children, preventive decision-making, and health information processing in early childhood contexts. In addition, longitudinal and interdisciplinary studies are needed to better understand how parental health literacy influences child health outcomes over time.

Improved conceptualization and measurement of parental health literacy may facilitate the identification of critical issues for health promotion and health education interventions targeting parents, particularly during early childhood. In the longer term, strengthening parental health literacy has the potential to reduce the burden on healthcare systems by decreasing the inappropriate or avoidable use of emergency pediatric services. The findings also support the integration of parental health literacy development into early childhood education and primary healthcare programs, with particular attention to preventive health behaviors and parent-focused guidance. Overall, these efforts may contribute to improved health outcomes for the next generation and support the reduction in health inequalities.

## Figures and Tables

**Table 1:** Search strategy: key words and related terms.

Key Words	Related Terms
health literacy	-
parent	caregiver, mother, father
child	-
measure	instrument, tool, questionnaire, survey, interview

Note. Search terms within the columns were combined with the Boolean operator “OR,” then searches on key words were combined using “AND.”.

**Table 2:** Inclusion and exclusion criteria for the systematic review.

Inclusion Criteria	
Year	from 2015 to 2024
Document type	Article
Source type	Journal
Review process	Peer-reviewed
Study type	Empirical
Content	Studies on health literacy among parents raising children under five; *and* Related to children’s health outcomes or parent caregiving practices.
Publication stage	Final
Language	English
**Exclusion criteria**	
Types of paper	systematic or other reviews, study protocols, pilot studies, policy papers
Content	Validation of measuring tools without any results; *or* No results related to parental health literacy; *or* Parental health literacy was examined, but not in relation to childcare or health needs/outcomes; *or* Studies related to children with special educational needs or with chronic diseases;
Access type	Full text unavailable

**Table 3:** General characteristics of the included studies.

Author (Year)	Country	Title	Journal Source	HL Domain	Care Domain or Health Outcome
Alqudah et al. (2019) [26]	Australia	Child fever management: A comparative study of Australian parents with limited and functional health literacy	Nursing and Health Sciences	general HL	Fever management
Amit Aharon et al. (2017) [27]	Israel	Parents with high levels of communicative and critical health literacy are less likely to vaccinate their children	Patient Education and Counseling	general HL	Vaccination
Brega et al. (2016) [25]	USA	Association of parental health literacy with oral health of Navajo Nation preschoolers	Health Education Research	oral HL	Oral health
Cormier et al. (2020) [28]	USA	eMental Health Literacy and Knowledge of Common Child Mental Health Disorders among Parents of Preschoolers	Issues in Mental Health Nursing	general eHL	Mental health
Graus et al. (2021) [29]	Germany	Breastfeeding behavior is not associated with health literacy: evidence from the German KUNO-Kids birth cohort study	Archives of Gynecology and Obstetrics	general HL	Breastfeeding behavior
Heerman et al. (2018) [30]	USA	Validity of the toddler feeding questionnaire for measuring parent authoritative and indulgent feeding practices which are associated with stress and health literacy among Latino parents of preschool children	Nutrition Research	general HL	Feeding practices
Hosseini et al. (2019) [31]	Iran	Investigating the Relationship Between Breastfeeding Duration and Health Literacy in Primiparous Women Referring to Tehran Health Centers: An Application of Bayesian Poisson Regression Model	Journal of Biostatistics and Epidemiology	general HL	Breastfeeding
Johri et al. (2015) [32]	India	Association between maternal health literacy and child vaccination in India: A cross-sectional study	Journal of Epidemiology & Community Health	general HL	Vaccination
Lee et al. (2018) [11]	USA	Exploring the relationship between maternal health literacy, parenting self-efficacy, and early parenting practices among low-income mothers with infants	Journal of Health Care for the Poor and Underserved	general HL	Early parenting practices
Liechty et al. (2015) [33]	USA	Health literacy and parent attitudes about weight control for children	Appetite	general HL	Weight control
Mekhail et al. (2024) [34]	Sweden	Parents’ comprehensive health literacy and child health after attending extended home visiting in Swedish multicultural settings-A case-comparison study.	Scandinavian Journal of Caring Science	general HL	Child health
Menekşe et al. (2024) [35]	Turkey	Determination of the relationship between parents’ health literacy and fever management of their children: A cross-sectional study	Journal of Advanced Nursing	general HL	Fever management
Menoncin et al. (2023) [36]	Brasil	Parental oral health literacy influences preschool children’s utilization of dental services	Brasilian Oral Research	oral HL	Dental health
Meppelink et al. (2019) [37]	Netherland	I was Right about Vaccination”: Confirmation Bias and Health Literacy in Online Health Information Seeking	Journal of Health Communication	general HL	Vaccination
Northrup and Smaldone (2017) [38]	USA	Maternal Attitudes, Normative Beliefs, and Subjective Norms of Mothers of 2- and 3-Year-Old Children	Journal of Pediatric Healthcare	general HL	Feeding practices/obesity
Pawellek et al. (2024) [39]	Germany	Effect of mothers‘ health literacy on early childhood allergy prevention behaviours: results from the KUNO-Kids health study	BMC Public Health	general HL	Early childhood allergy prevention (ECAP)
Soe et al. (2024) [40]	Myanmar	Hygiene practice and diarrhea prevalence among underfive children in Myanmar: a cross-sectional study.	BMC Pediatrics	hygiene health literacy	Diarrhea prevalence
Welkom et al. (2016) [41]	USA	Associations between Caregiver Health Literacy and Preschool Children’s Secondhand Smoke Exposure	Journal of Pediatric Psychology	general HL	Second-hand Smoke Exposure

**Table 4:** Methodological characteristics of the included studies.

Author (Year)	Study Aim	Study Design	Study Population	Research Tool	Findings
Alqudah et al. (2019) [26]	To compare parents/carers’ knowledge of fever management for a child in parents with limited or functional HL	cross-sectional measurement performed as part of an intervention	155 parents	REALM-SF; Fever Knowledge Scale; Fever Management Practices Scale;	Regardless of their health literacy level, the parents participating in the study demonstrated limited knowledge and inadequate practices regarding fever recognition and management.
Amit Aharon et al. (2017) [27]	To investigate the relationship between parents’ health literacy and decision-making regarding child vaccinations.	cross-sectional survey	731 parents	Health Literacy Questionnaire developed by Ishikawa et al. (2008) [42]; Questionnaire about vaccination	Parents with high functional, communicative, and critical HL are more at risk of not vaccinating their children.
Brega et al. (2016) [25]	To explore the association between parental health literacy and children’s oral health outcomes among families on the Navajo Nation.	cross-sectional measurement performed as part of an intervention	1061 child–parent dyads	Modified version of Chew et al.’s (2004) [43] three health literacy screening items	Parents with more limited health literacy had significantly worse oral health status and reported their children to have significantly worse oral health-related quality of life.
Cormier et al. (2020) [28]	To explore and assess the relationships between parents’ eMental health literacy skills and their knowledge of common child mental health disorders and their child difficulties.	cross-sectional survey	151 parents	Modified eHealth literacy scale (eHEALS); Strength and Difficulties Questionnaire (SDQ) P 2–4;	Children of parents with low eMental HL were more likely to be rated as having a high or very high risk of a mental health disorder compared to those, whose parents had high eMental HL
Graus et al. (2021) [29]	To investigate the role of maternal health literacy in breastfeeding behavior.	longitudinal cohort study	1172 mother–child dyads	HLS-EU-Q47	The study found no connection between health literacy and breastfeeding behavior.
Heerman et al. (2018) [30]	To validate Toddler Feeding Questionnaire (TFQ) in a large Latino sample To examine whether parental characteristics such as BMI, stress, and health literacy are linked to more indulgent and less authoritative feeding practices.	cross-sectional measurement performed as part of an intervention	555 parent–child pairs	Newest Vital Sign; Perceived Stress Scale; Toddler Feeding Questionnaire;	High parental stress and low health literacy are associated with unhealthy feeding practices and an increased risk of childhood obesity.
Hosseini et al. (2019) [31]	To investigate the factors affecting breastfeeding duration in primiparous women.	cross-sectional survey	190 mothers	Health Literacy for Iranian Adults (HELIA)	A significant correlation was shown between health literacy score and duration of breastfeeding.
Johri et al. (2015) [32]	To test the hypothesis that maternal health literacy is positively associated with children’s receipt of vaccines.	cross-sectional survey	1170 rural mothers, 670 urban mothers	self-developed measurement tool related to parents’ HL and children’s vaccination	Maternal health literacy is associated with child vaccination. Initiatives targeting health literacy could improve vaccination coverage.
Lee et al. (2018) [11]	To explore the association of maternal health literacy (MHL), parenting self-efficacy and early parenting practices among low-income mothers with infants.	cross-sectional descriptive study	186 mothers with infants	Newest Vital Sign; Early Parenting Practices Index (EPPI);	Parenting self-efficacy has a mediating effect on MHL and early parenting practices among mothers with infants.
Liechty et al. (2015) [33]	To examine associations between parental health literacy and parent attitudes about weight control strategies for young children	cross-sectional study conducted as part of a longitudinal panel study	497 parents	Newest Vital Signs; Health Information National Trends Survey; Child Feeding Questionnaire (CFQ);	Health literacy influences parental views and attitudes about child Low health literacy may also be a risk factor for selecting more unsafe weight control strategies for children.
Mekhail et al. (2024) [34]	To gain knowledge about associations between parents’ comprehensive HL (CHL) and child health	quasi-experimental study used a case–control sampling	151 parents	HLS-EU-Q16; Data collection from children’s medical records	The study found no significant association between parents’ CHL and children’s health outcomes, including breastfeeding and exposure to tobacco smoke.
Menekşe et al. (2024) [35]	To determine the relationship between the HL of Turkish parents and fever management of their children.	Cross-sectional study	242 parents	Parent Descriptive Information Form; Turkish HL Scale-32; Parents’ Fever Management Scale	The fever management of parents with higher HL levels is regarded more appropriate.
Menoncin et al. (2023) [36]	To evaluate the impact of parental OHL on the use of dental services by Brazilian preschoolers	cross-sectional study	419 parents	Oral Health Literacy Adult Questionnaire (OHL-AQ)	Parents with lower oral hygiene knowledge are less likely to ensure their children attend preventive dental visits.
Meppelink et al. (2019) [37]	To examine the role of confirmation bias in information seeking related to early-childhood vaccination.	online cross-sectional survey	480 parents	Newest Vital Sign	Parents tend to preferentially seek information that confirms their pre-existing beliefs, rather than information that contradicts them, when engaging in online searches related to early childhood vaccination.
Northrup and Smaldone (2017) [38]	To examine maternal attitudes, normative beliefs and meal selection behaviors of mothers with young children	cross-sectional study	31 mothers	Newest Vital Sign; Diet and Health Knowledge Survey–Short Form; Body Outline Silhouette; Feeding simulation exercise (FSE)	Mothers’ knowledge (normative beliefs) was overall poor regarding USDA recommendations for their children.
Pawellek et al. (2024) [39]	To examine the causal effect of mothers’ HL on early childhood allergy prevention behaviors and to assess potential moderators of this effect.	longitudinal cohort study	1662 mothers	HLS-EU-Q47; ECAP:allergy risk status EBI (“Eltern-Belastungs-Inventar” = parenting stress index) PHQ-D (Patient Health Questionnaire)	Lower maternal health literacy contributed to an increased tendency to adopt allergen-avoidant behaviors in early childhood, while diminishing the likelihood of exclusive breastfeeding.
Soe et al. (2024) [40]	To identify the association between hygiene practices in families and childhood diarrhea among young children	cross-sectional study	1207 families	Self-designed questionnaire: HL domains: sanitation promotion, hygiene promotion, diarrhea prevention; Observation of hygiene facilities and practices	Father’s handwashing practices and hygiene promotion health literacy were significant determinants of childhood diarrheal outcomes.
Welkom et al. (2016) [41]	To explore the relationship between caregivers’ health literacy and (1) their expectations about the consequences of smoking, (2) their implementation of home and car smoking bans, and (3) their children’s exposure to second-hand smoke.	cross-sectional study conducted as part of a longitudinal panel study	268 parents	REALM-SF; Smoking-Related Outcome Expectancies; Home air nicotine and child salivary cotinine were collected as indicators of child second-hand smoke exposure.	Caregivers’ HL is associated with child second-hand smoke exposure and is important in shaping smoking-related beliefs.

## Data Availability

No new data were created or analyzed in this study.

## Supplementary Materials

The following supporting information can be downloaded at: https://www.mdpi.com/article/10.3390/children13050685/s1, Table S1: Summary of Risk of Bias in cross-sectional studies [11,21,25,26,27,28,30,31,32,33,35,36,37,40,41]; Table S2: Summary of Risk of Bias in cohort studies [22,29,39]; Table S3: Summary of Risk of Bias in quasi-experimental studies [23,34,38].
