# Supplementary material for: Parental Health Literacy as a Determinant of Parenting Practices and Early Childhood Health Outcomes: A Systematic Review
Source: Children (Basel). 2026 May 16;13(5):685. doi: 10.3390/children13050685 (PMC13204775; doi:10.3390/children13050685)
Supplement: Supplementary file 1 [file children-13-00685-s001.zip › children-4309567-supplementary.pdf]

Table S1. Summary of Risk of Bias in cross-sectional studies [21]

Author (Year)	Study design	Selection				Comparability	Outcome		
		Representa-tiveness of the sample	Sample size	Non-Respondents/ missing data	Ascertainment of the exposure (risk factor)	Comparability of subjects in different outcome groups on the basis of design or analysis. Confounding factors controlled.	Assessment of the outcome	Statistical test	Overall rating
**Alqudah et al (2019) [26]**	cross-sectional study as part of an intervention	-	*	-	*	**	*	*	Satisfactory Study
**Amit Aharon et al (2017) [27]**	cross-sectional	-	*	-	**	*	*	*	Satisfactory Study
**Brega et al (2015) [25]**	cross-sectional study as part of an intervention	*	*	-	*	**	*	*	Good study
**Cormier et al (2020) [28]**	cross-sectional design	-	-	-	**	*	*	*	Satisfactory Study
**Heerman et al (2018) [30]**	cross-sectional study as part of an intervention	*	-	-	*	**	*	*	Satisfactory Study
**Housseini et al (2019) [31]**	cross-sectional	*	-	-	**	*	*	*	Satisfactory Study
**Johri et al (2015) [32]**	cross-sectional	*	*	*	*	*	*	*	Good study
**Lee et al (2018) [11]**	cross-sectional descriptive study	-	-	-	*	**	**	*	Satisfactory Study
**Liechty et al (2015) [33]**	cross-sectional study as a part of a longitudinal study	*	-	-	*	**	**	*	Good study

**Menekşe et al (2024) [35]**	cross-sectional study	*	*	-	**	**	**	*	Very good study
**Menoncin et al (2023) [36]**	cross-sectional study	*	*	*	*	**	**	*	Very good study
**Meppelink et al (2019) [37]**	cross-sectional study	-	-	-	*	**	**	*	Satisfactory Study
**Soe et al. (2024) [40]**	cross-sectional study	*	*	-	*	**	**	*	Good study
**Welkom et al (2016) [41]**	cross-sectional study as part of longitudinal study	*	*	*	*	**	**	*	Very good study

Note: ** (two stars): the study met a higher-quality standard for that criterion; * (one star): the criterion was justified or satisfactory; - (dash): the criterion was not fulfilled, was unclear, or insufficient information was provided.
Evaluation: very good studies: 9-10 points, **good studies:** 7-8 points, **satisfactory studies:** 5-6 points, **unsatisfactory studies:** 0 to 4 points.

Table S2. Summary of Risk of Bias in cohort studies [22]

**Author (Year)**	**Study design**	**Selection**				**Comparability**	**Outcome**			**OverallQuality**
		**Representative-ness of the exposed cohort**	**Selection of the non-exposed cohort**	**Ascertain-ment of exposure**	**Demonstration that outcome of interest was not present at start of study**	**Comparability of cohorts on the basis of the design or analysis controlled for confounders**	**Assessment of outcome**	**Was follow-up long enough for outcomes to occur**	**Adequacy of follow-up of cohorts**	
**Graus et al (2021) [29]**	longitudinal cohort study	**-**	**-**	*	*	*	*	*	**-**	**Fair**
**Pawellek et al (2024) [39]**	observational, prospective cohort study	**-**	**-**	*	*	*	*	*	**-**	**Fair**

Note: * (star): the study met the quality requirement for that item; - (dash): the criterion was not fulfilled, was unclear, or insufficient information was provided.
Evaluation: good quality: 3 or 4 stars in selection domain AND 1 or 2 stars in comparability domain AND 2 or 3 stars in outcome/exposure domain; **fair quality:** 2 stars in selection domain AND 1 or 2 stars in comparability domain AND 2 or 3 stars in outcome/exposure domain; **poor quality:** 0 or 1 star in selection domain OR 0 stars in comparability domain OR 0 or 1 stars in outcome/exposure domain.

Table S3. Summary of Risk of Bias in quasi-experimental studies [23]

Bias	Questions	Mekhail et al (2024) [34]				Northrup & Smaldone (2017) [38]			
		yes	no	unclear	N/A	yes	no	unclear	N/A
**Bias related to temporal precedence**	It is clear in the study what is the "cause" and what is the "effect" (ie, there is no confusion about which variable comes first)?	x				x			
**Bias related to selection and allocation**	Was there a control group?	x					x		
**Bias related to confounding factors**	Were participants included in any comparisons similar?				x				x
**Bias related to administration of intervention/exposure**	Were the participants included in any comparisons receiving similar treatment/care, other than the exposure or intervention of interest?		x			x			
	Were there multiple measurements of the outcome, both pre and post the intervention/exposure?	x				x			
**Bias related to assessment, detection, and measurement of the outcome**	Were the outcomes of participants included in any comparisons measured in the same way?			x		x			
	Were outcomes measured in a reliable way	x				x			
**Bias related to participant retention**	Was follow-up complete and if not, were differences between groups in terms of their follow-up adequately described and analyzed?	x							x
**Statistical Conclusion Validity**	Was appropriate statistical analysis used?	x				x			
